# Tracking of Dietary Intake and Diet Quality from Late Pregnancy to the Postpartum Period

**DOI:** 10.3390/nu11092080

**Published:** 2019-09-03

**Authors:** Audrée Lebrun, Anne-Sophie Plante, Claudia Savard, Camille Dugas, Bénédicte Fontaine-Bisson, Simone Lemieux, Julie Robitaille, Anne-Sophie Morisset

**Affiliations:** 1 School of Nutrition, Laval University, Québec City, QC G1V 0A6, Canada; 2 Endocrinology and Nephrology Unit, CHU de Québec-Université Laval Research Center, Québec City, QC G1V 4G2, Canada; 3 Institute of Nutrition and Functional Foods (INAF), Laval University, Québec City, QC G1V 0A6, Canada; 4 School of Nutrition Sciences, University of Ottawa, Ottawa, ON K1N 6N5, Canada; 5 Institut du Savoir Montfort, Montfort Hospital, Ottawa, ON K1K 0T2, Canada

**Keywords:** pregnancy, postpartum, dietary intake, energy intake, supplements, dietary reference intakes (DRIs), diet quality, Healthy Eating Index

## Abstract

The present study aimed to characterize dietary intake and diet quality from late pregnancy to six months postpartum. Participants (*n* = 28) completed 2–3 Web-based 24 h recalls at three distinct periods: (1) during the third trimester of pregnancy; (2) three months and (3) six months after delivery. Energy, macro-and micronutrient intakes (from foods and supplements), as well as the Canadian healthy eating index (C-HEI) were derived from the dietary recalls. No significant variation in energy and macronutrient intakes was observed between time points. The proportion of women taking at least one supplement decreased over time (*p* = 0.003). The total intake of several micronutrients (vitamins A, C, D, group B vitamins, iron, magnesium, zinc, calcium, phosphorus, manganese, and copper) decreased significantly over time (*p* < 0.05 for all micronutrients). The total C-HEI score and its components did not change, except for the total vegetables and fruit subscore, which decreased over time (8.2 ± 2.0 in the 3rd trimester, 7.1 ± 2.2 at three months postpartum, 6.9 ± 2.4 at 6 months postpartum, *p* = 0.04). In conclusion, we observed a general stability in diet quality, energy, and macronutrient intakes from the third trimester of pregnancy to six months postpartum. However, several micronutrient intakes decreased over time, mostly due to changes in supplement use.

## 1. Introduction

Adopting healthy eating behaviors is crucial during pregnancy in order to positively influence both the mother’s and the child’s health [1,2,3]. Maintaining a balanced diet after childbirth is also important to ensure optimal maternal health, both in the short and long term [4,5,6,7]. In the short term, a woman’s diet after delivery can influence weight retention since it is associated with the total energy intake [4]. In the long term, postpartum weight retention has been identified as a contributor to obesity, the latter being associated with an increase in morbidity and mortality risk [5,6]. Furthermore, adequate diet quality and dietary intake are essential to support the energy demand associated with lactation and ensure optimal early life nutrition for the newborn. In fact, greater maternal diet quality during pregnancy and lactation has been inversely associated with infant weight and adiposity in the early postpartum period, which could prevent obesity later in life [7]. Hence, it is important to maintain healthy eating behaviors both during and after pregnancy.

However, few studies have investigated this continuum and the changes that can occur from pregnancy to the postpartum period. One study showed that Swedish women’s diet quality tended to decrease after delivery, mostly due to an increased intake of discretionary food (e.g., sweets, cakes, cookies, crisps, and ice cream), and a decreased intake of vegetables and fruit [8]. Although a different study found a significant increase in the proportion of women engaging in more positive behaviors (drinking two or more cups of milk per day, consuming three or more servings of vegetables and fruit per day, and eating breakfast every day) from pre-pregnancy to pregnancy, that proportion decreased dramatically at six months postpartum [9]. It was also reported, in a cohort of low-income women with diverse ethnicity, that following childbirth, mean daily servings of grains, vegetables and fruit declined, while the percentage of energy from fat and added sugar increased in comparison with pregnancy [10]. Overall, although an improvement in diet quality has been reported during pregnancy [9,11], healthy eating habits adopted in the prenatal period are not often maintained after childbirth [8,9,10,11,12].

Nevertheless, studies examining maternal diet from late pregnancy to the postpartum period rarely detail women’s adherence to dietary recommendations [8,9,10,11,12], despite the significant impact of nutrition on maternal and child health. Thus, it appears relevant to examine women’s diet during their transition to maternity as well as look at their adherence to nutritional recommendations. The aims of this study are to characterize dietary intake and diet quality from late pregnancy to the postpartum period and to investigate women’s adherence to current Canadian nutritional recommendations at each time point. Firstly, we hypothesize that diet quality decreases from the third trimester to six months postpartum. Secondly, we hypothesize that adherence to micronutrient intake recommendations will be low in the postpartum period, especially for lactating women in whom nutritional needs are increased.

## 2. Materials and Methods

### 2.1. Study Population

**Figure 1:**
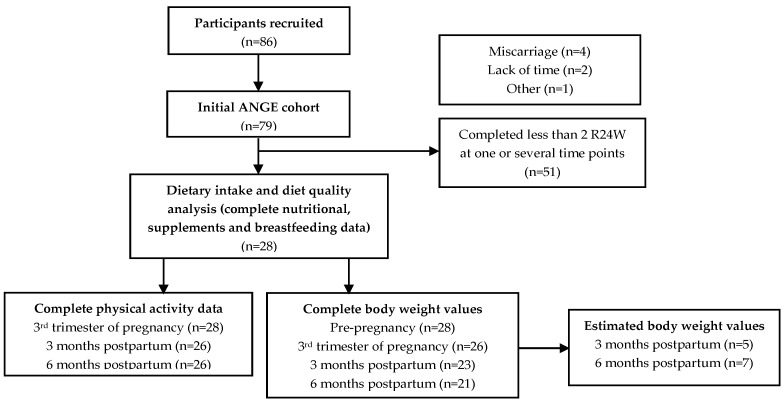
Flowchart of the study sample.

The ANGE (Apports Nutritionnels durant la GrossessE) project included eighty-six (86) pregnant women recruited in Quebec City, QC, Canada. At the recruitment of the initial cohort, the exclusion criteria were to be under 18 years of age or to present a severe medical condition (i.e., type 1 or 2 diabetes, renal disease, inflammatory and autoimmune disorders). Previously published analyses included 79 women with complete nutritional data at each trimester of pregnancy [13,14]. In the present paper, the final sample includes 28 women for whom we have complete nutritional information for the 3rd trimester of pregnancy, as well as for the 3- and 6-months postpartum time points (see Figure 1). The Institutional Ethics Committee approved the project (Reference number: 2016–2866) and participants gave their informed written consent.

### 2.2. The Automated Web-Based 24 h Recall (R24W)

In the 3rd trimester of pregnancy (range: 31.9–36.1 gestational weeks, gestational age confirmed by ultrasound in the 1st trimester), at 3 months postpartum (range: 9.4–13.7 weeks after delivery) and 6 months postpartum (range: 23.0–26.4 weeks after delivery), participants used the R24W (Rappel de 24 h Web) platform. They completed, at each period, 2–3 Web-based 24 h dietary recalls. The average time between recalls was 1.0 ± 0.5, 1.4 ± 0.9 and 1.3 ± 0.7 weeks in the 3rd trimester, at 3 months postpartum, and at 6 months postpartum, respectively. The R24W has been previously described and was validated in pregnant women as well as in the general adult population [15,16,17]. Data regarding intake of total energy and 22 nutrients were analyzed.

### 2.3. Healthy Eating Index

The R24W platform automatically calculates the 2007 version of the Canadian Healthy Eating Index (C-HEI), an adaptation of the HEI developed by Kennedy et al. [18,19] that was used to assess diet quality at each time point (3rd trimester, 3rd and 6th month of postpartum). The HEI has been validated in the general population and used by various authors to assess diet quality among pregnant women [18,20,21,22]. The assessment of the C-HEI relies on the number of servings reported by an individual, according to age and sex, as specified in the 2007 version of Canada’s Food Guide [18,23]. In brief, the C-HEI is divided into eight adequacy components and three moderation components [18]. Based on the scoring criteria, participants were allocated a score between 0 and the potential maximum (5, 10, or 20). In accordance with the method described earlier [18,23], scores are then added up for a maximum of 100 points, representing a perfect adherence to the 2007 version of Canada’s Food Guide (see detailed method in Supplementary Table S1).

### 2.4. Supplement Use

All participants completed, at each time point, a Web questionnaire collecting information on supplement use. This questionnaire was previously described elsewhere [14]. In brief, for each supplement they reported taking, participants had to provide its name, its drug identification number, its measurement unit (e.g., tablet, drop, gram, milliliter, etc.) and its dose, as well as the frequency at which the reported dose was taken (e.g., once a day, twice a week, etc.). Nutritional data for all supplements were obtained by using the Health Canada Licensed Natural Health Product Database or the companies’ product labels [24]. A research assistant reached out to participants when any supplement’s characteristic was missing or incomplete. The use of supplements was compiled according to the supplement’s type (multivitamin or one-nutrient supplement) and the number of women that reported taking each supplement.

### 2.5. Other Web Questionnaires

Women completed questionnaires on socioeconomic status and general health early in pregnancy to obtain data on sample descriptive characteristics. A Web-based self-administered questionnaire was completed by 27 women at 3 and 6 months postpartum to report the infant feeding methods and a research assistant contacted women with missing information.

### 2.6. Estimated Energy and Protein Requirements

Pre-pregnancy BMI was calculated using self-reported pre-pregnancy weight and measured height. The validated version of the pregnancy physical activity questionnaire (PPAQ) was completed by women as well as the International physical activity questionnaire (IPAQ) at 3 and 6 months postpartum [25,26,27]. Physical activity levels (PALs) were calculated with the total number of minutes per day participants engaged in moderate and high-intensity activities. Based on the dietary reference intakes (DRIs), we characterized participants as either sedentary, low-active, active or very active [28]. Estimated energy requirements (EERs) in the 3rd trimester were calculated with the participant’s age, height, pre-pregnancy weight, physical activity level, to which 452 kcal was added [28]. The weight of 26 women was measured during an on-site visit at the 3rd trimester of pregnancy. In the postpartum periods, a Web-based self-administered questionnaire regarding maternal weight was completed by 23 women at 3 months and by 21 women at 6 months. Mean weight difference between each period and pre-pregnancy was calculated (see Table 1). The difference between postpartum periods and pre-pregnancy weights can then be defined as weight retention. Since body weight values were missing for 5 women at 3 months postpartum and for 7 women at 6 months postpartum (see Figure 1), an estimation of their weight difference (in kg) was made at those time points, in order to calculate their estimated energy and protein requirements. The estimated weight difference was calculated by using the mean weight difference (vs. pre-pregnancy weight) for the 23 women at 3 months (4.3 kg) and the mean weight difference for the 21 women at 6 months (2.8 kg). We were therefore able to calculate EERs at each postpartum period by using age, height, weight, physical activity levels and by adding 500 kcal only for women who were breastfeeding at the time they completed the questionnaires [28]. In the 3rd trimester, daily estimated protein requirements (EPRs) were determined with the following calculation: 1.1 g/kg of pre-pregnancy weight + 25 g of protein [28]. For postpartum periods, daily EPRs were estimated as 1.3 g/kg of reported or estimated weight in breastfeeding mothers and 0.8 g/kg for others [28].

### 2.7. Statistical Analyses

Based on the automatically generated data from the completed dietary recalls, means and standard deviations were determined for energy, macro- and micronutrient intakes at each time point. Micronutrient intake from supplements and from food sources were compiled to estimate the total micronutrient intake. The intake of energy and nutrients were compared to their respective dietary reference intakes (DRIs). We then obtained a number of women that had intakes below the estimated average intakes (EARs) or above the upper intake limit (UL), when relevant [29]. A comparison was made between folate intake expressed as dietary folate equivalents (DFE) and the folate EAR. Comparison with the folic acid UL was only made with fortified foods and supplements because this limit is only applicable to synthetic forms of folic acid [28]. Similarly, only intake from supplements were compared to the UL for niacin and magnesium intakes [28]. The average energy intake (EI) and percentages of energy provided by each macronutrient (protein, carbohydrate, fats) were compared respectively with EERs and the acceptable ranges for each macronutrient [28]. The acceptable macronutrient distribution ranges (AMDR) are recommended intervals for energy proportion provided from each macronutrient, in order to ensure an adequate intake of essential nutrients [28]. Percentages of women with data below or above their EERs or AMDR were determined. Comparison of protein intake (g/day) with the EPRs was performed [28]. To assess changes in energy, macro- and micronutrient intakes and HEI scores from the 3rd trimester of pregnancy to 6 months postpartum, repeated measure analyses of variance (ANOVA) were computed. Tukey’s honest significant difference post-hoc tests were then performed to identify specific differences between time points. Chi-square tests were conducted to compare supplement use (categorical variables) over time. All statistical analyses were performed with JMP version 14 (SAS Institute Inc., Cary, NC, USA).

## 3. Results

Participants’ characteristics are presented in Table 1. All 28 included women were Caucasians and on average in their early thirties. The sample covered a wide range of pre-pregnancy BMI, but most of the participants had a normal weight. The majority was also multiparous and had a high level of education as well as a substantial household income. Most women breastfed, exclusively or not, up to six months after delivery. This sample’s characteristics (*n* = 28) do not differ significantly from the ANGE project’s original sample (*n* = 79) [13].

### 3.1. Energy and Macronutrients

Table 2 shows that no significant variation was observed for energy and macronutrient intakes across time points. Energy intake was below EER for 61%, 78% and 74% of the participants in the 3rd trimester of pregnancy, at three months and at six months postpartum, respectively. When macronutrient intake was examined in grams (Supplementary Table S2), protein intake of at least 64% of the participants exceeded the EPR at each period. However, at each time points, all women had protein intake as a percentage of energy within the AMDR. At each time point, more than 57% of women reported fat intake as percentages of energy intake that was above the AMDR. Inversely, carbohydrate intake as percentages of energy was below the AMDR range for up to 50% of participants. Dietary fiber intake decreased over time (*p* = 0.01) and more than 89% of participants had intake below the DRI at each period (Supplementary Table S2). No significant difference in energy and nutrient intakes was observed between normal weight, overweight or obese participants (data not shown). Secondary analyses were conducted to test associations between energy intake and weight retention (data not shown). No significant difference in weight retention was observed between participants who were below or over their EERs. However, Spearman’s correlation showed that energy intake at six months after delivery was positively associated with postpartum weight retention at the same period (r = 0.52, *p* = 0.02).

### 3.2. Vitamins and Minerals

Micronutrient intake derived from the R24W (food sources only) and proportions of women that reported intake above or below the corresponding DRIs are presented in Table 3. Micronutrient intake from food alone decreased from late pregnancy to six months postpartum for vitamins A and C as well as thiamin, riboflavin, calcium, and phosphorus (*p* ≤ 0.03 for all micronutrients). A downward trend was observed for vitamin D, vitamin B_12_, and iron intakes. As presented in Table 4, fewer women reported the use of supplements after delivery (89%, 68% and 46% at the 3rd trimester of pregnancy, at three and six months postpartum, respectively, *p* = 0.003), and multivitamins remained the most commonly used supplement. A secondary analysis showed that 67% and 52% of participants who breastfed were taking at least one supplement at three and six months after delivery, respectively. As shown in Table 5, when food sources and dietary supplements were combined, total micronutrient intake decreased from late pregnancy to the postpartum period for vitamins A, B_6_, B_12_, C and D as well as for thiamin, riboflavin, niacin, pantothenic acid, iron, magnesium, zinc, calcium, phosphorus, manganese and copper (*p* ≤ 0.03 for all micronutrients). At each period, a higher proportion of women had a total micronutrient intake below the EAR, particularly for vitamins A (4%, 54%, 54%) and D (11%, 36%, 57%). Total intake of sodium was above the UL for more than 79% of women at each time point. Similar results regarding the total micronutrient intake as well as EAR and UL adherence were observed, both at three and six months postpartum, when looking only at lactating women, which represents 89%–96% of our sample.

### 3.3. Diet Quality

Mean HEI scores are presented in Table 6. Total and sub-scores did not significantly vary from late pregnancy to six months postpartum, except for the total vegetables and fruit sub-score, which decreased over time (*p* = 0.04) and more specifically between the third trimester of pregnancy and six months postpartum (*p* = 0.03).

## 4. Discussion

Our prospective evaluation of women’s dietary intake revealed stability in energy and macronutrient intakes from late pregnancy to six months postpartum. Most women were below their energy estimated requirements and above their protein estimated requirements. Total micronutrient intake decreased from late pregnancy to six months after delivery for many vitamins and minerals. We also observed a decrease in diet quality regarding the total consumption of vegetables and fruit.

Stability in energy intake was found from late pregnancy to six months after delivery. Likewise, Talai Rad et al., as well as Moran et al. found no significant variation in women’s energy intake from pregnancy to the postpartum period [20,30]. In contrast, George et al. found that the transition from pregnancy to the postpartum period was associated with a decrease in the mean energy intake in the overall sample, and in both lactating and nonlactating low-income women [10]. However, our small study sample consisted mostly of lactating women, for whom the energy estimated requirements were similar from late pregnancy to the postpartum period, which may explain the stability in energy intake. We also found that most women were under their respective EERs in the third trimester of pregnancy as well as in the postpartum period. In contrast, Moran et al. found that most of 301 overweight or obese women met the Australian Nutrient Reference Values in energy from pregnancy to four months postpartum [20]. In comparison with our participants, women in this study were all overweight or obese and came from an area of greater social deprivation [20]. Also, at four months after delivery, 57% of Australian women were breastfeeding versus 96% of our participants at three months postpartum [20]. Considering that breastfeeding requires additional energy intake, we suggest that lactating mothers encounter more difficulties in meeting these caloric recommendations. The underreporting of energy intake may also have influenced mean caloric intake of our study sample considering that other studies have reported divergent percentages (between 13% and 49%) of under-reporters during pregnancy [31,32,33,34]. However, there is a lack of consistency in the methods and thresholds used to evaluate the misreporting of energy intake in pregnancy, indicating a need to further investigate which method would be the most appropriate to use.

Regarding macronutrient intake, most of our participants had protein intake (as percentages of energy intake) within their respective acceptable distribution ranges, at all time points. However, the majority of our participants had protein intake that exceeded their EPRs at all periods, similarly to Moran et al. [20]. Further, up to 50% of women had carbohydrate consumption (as a percentage of energy intake) below the AMDR, with a higher proportion in the postpartum period compared to what has been observed in overall pregnancy [14]. Furthermore, many participants had fat intake as a percentage of energy intake that was above the acceptable distribution range from late pregnancy to six months after delivery. Similarly, Talai Rad et al. found that in 32 healthy women, the proportion of fat intake within the total caloric intake (36%) slightly exceeded the German Nutrition Society recommendation from early pregnancy to six weeks after delivery [30]. Additionally, a previous analysis in this cohort found that macronutrient intake, as percentages of energy intake, was stable throughout pregnancy [14]. Hence, the mean intake of macronutrients, in comparison with acceptable distribution ranges, does not seem to change from early pregnancy to six months after delivery in our cohort of high-income women. Finally, we observed that almost all women did not meet the adequate intake recommended for dietary fiber, at each period, similarly to the results obtained during pregnancy in the initial ANGE cohort [14] and in the general population [35]. Also, fiber intake decreased from late pregnancy to six months after delivery, in contrast to Moran et al. who found a stability from pregnancy to four months postpartum [20].

Despite our small sample size, we found that total intake of 16 of the 20 vitamins and minerals had significantly decreased from the third trimester of pregnancy to six months postpartum, which is concordant with the decrease in vegetables and fruit intakes observed with the HEI-score. In addition, most participants did not meet the recommendation for vitamin A in the postpartum period since the EAR for this vitamin almost doubles in the context of lactation compared to pregnancy [29]. Also, a significant proportion of women failed to meet the recommendation for vitamin D after delivery. A decreasing trend observed for the milk and alternatives HEI subscore may partially explain the observed decrease in vitamin D since most of these foods are fortified with vitamin D. Nevertheless, and more importantly, the decrease in micronutrient intake might be explained by the decline in supplement use following the delivery, despite the Society of Obstetricians and Gynaecologists of Canada’s recommendation for women to keep taking a prenatal multivitamin as long as breastfeeding continues [36]. Similar results regarding total micronutrient intake and adherence to recommendations were observed in the postpartum period when looking only at lactating women, which represent most of our sample. A decrease in total micronutrient intake of breastfeeding participants persisted even if more than half of them continued taking at least one supplement after delivery. Moran et al. also found lower total intake of iron, zinc and calcium as well as vitamins A, B_6_ and C from the third trimester to four months postpartum, with a significant decrease in supplement use over this period [20]. It would therefore appear that women reduced their supplement use after childbirth, which may put lactating women at risk of non-adherence to micronutrient recommendations, since their nutritional needs are increased compared to non-lactating women.

Diet quality remained stable from late pregnancy to the postpartum period in this limited sample size, except for the total vegetables and fruit sub-score. Total C-HEI score and its components are based on the number of servings consumed, therefore participants seemed to decrease their vegetable and fruit intakes after delivery in comparison to late pregnancy. This might have had an impact on the observed decrease in dietary intake of fiber, vitamin A, and vitamin C. As previously published by our research team (initial ANGE cohort), total C-HEI scores did not significantly vary throughout pregnancy [13]. However, the adequacy sub-score decreased significantly from early to late pregnancy, mostly due to a decreased intake in vegetables and fruit [13]. We can hypothesize that vegetables and fruit intakes decrease throughout pregnancy, which continues in the postpartum period, as reported in other studies [8,9,10]. This is concordant with the supposition that motivation for healthy eating might decrease as pregnancy progresses and after delivery. Interestingly, a study found that multiparous women, who make up the majority of this sample, have lower intentions to eat in a healthier manner compared to new and non-parents [37]. The same authors hypothesized that mothers may find it difficult to put time and energy in preparing healthy meals for multiple children, thus leading to the subsequent decrease in motivation for their own dietary behaviors [37]. As a possible strategy to counter the decrease in micronutrient intake and diet quality, healthcare providers should reinforce recommendations regarding multivitamin supplementation and address the importance of vegetables and fruit consumption during postpartum follow-ups.

To our knowledge, this is the first study to prospectively assess whether women adhere or not to current Canadian nutritional recommendations up to six months after delivery. A major strength of this study is the use of detailed information collected on dietary intake with the completion of 2–3 validated Web-based 24 h recalls at each period combined with a Web questionnaire on supplements use. However, some limitations need to be acknowledged, mainly regarding the small sample size and lack of representativeness of our sample. The small sample size could have attenuated the statistical significance, however, the results we observed were similar to those from other studies in larger cohorts. Our sample can also include a potential proportion of under-reporters and a possible overestimation of the energy requirement of overweight or obese breastfeeding women [38], which would have inflated the proportion of women not meeting their EERs. Since all women were Caucasian and most of them were of higher socioeconomic status, our results may not be representative of mothers from a more ethnically and socioeconomically diverse population. Diet quality and dietary intake should be further investigated in a larger and more representative cohort, from pregnancy to the postpartum period.

## 5. Conclusions

In summary, we observed relative stability in diet quality, energy and macronutrient intakes from late pregnancy to the postpartum period, but intake of several micronutrients decreased from the third trimester of pregnancy to six months after delivery. Decreased use of supplements, energy intake below the estimated requirements, lower intake of vegetables and fruit as well as increased micronutrient needs may put lactating women at risk of nutritional deficiencies. However, further research is needed, first, to confirm in a larger and more diverse cohort the decrease we observed in micronutrient intake, as well as in vegetables and fruit consumption from pregnancy to the postpartum period, and second, to identify women at a higher risk of inadequate postpartum diet that could be targeted in future interventions.

## Figures and Tables

**Table 1:** Participants’ characteristics (*n* = 28).

Variables	Mean ± SD or *n* (%)
Age at study enrollment (years)	32.7 ± 3.6
Primiparous	12 (43)
Pre-pregnancy BMI (kg/m^2^)	24.9 ± 5.1
Normal weight	18 (64)
Overweight	6 (21)
Obese	4 (14)
Weight (kg)	
Pre-pregnancy	69.3 ± 16.6
3rd trimester of pregnancy (difference with pre-pregnancy) ^a^	12.8 ± 4.5
3 months postpartum (difference with pre-pregnancy) ^b^	4.3 ± 6.3
6 months postpartum (difference with pre-pregnancy) ^c^	2.8 ± 5.7
Ethnicity-Caucasian	28 (100)
Education	
College	2 (7)
University	26 (93)
Household income	
<60,000$	4 (14)
60,000–79,999$	5 (18)
80,000–99,999$	10 (36)
>100,000$	9 (32)
Breastfeeding (exclusively or not)	
At 3 months postpartum	27 (96)
At 6 months postpartum	25 (89)
Duration of exclusive breastfeeding postpartum (months) ^c^	5.2 ± 1.0

^a^*n* = 26; ^b^ *n* = 23; ^c^ *n* = 21.

**Table 2:** Period-specific energy and macronutrient intakes as percentages of energy intake in comparison with dietary reference intakes.

	3rd Trimester of Pregnancy			3 Months Postpartum			6 Months Postpartum			
	Mean ± SD	%Below AMDR, EER or UL	%Above AMDR, EER or UL	Mean ± SD	%Below AMDR, EER or UL	%Above AMDR, EER or UL	Mean ± SD	%Below AMDR, EER or UL	%Above AMDR, EER or UL	Overall *p*-Value ^b^
EER (kcal/day) ^a^	2497 ± 203	-	-	2578 ± 201	-	-	2678 ± 327	-	-	-
Energy intake (kcal/day)	2321 ± 429	61	39	2305 ± 506	78	22	2227 ± 474	74	26	0.48
AMDR protein, E%	10–35	-	-	10–35	-	-	10–35	-	-	-
Protein, E%	17.5 ± 2.8	0	0	17.1 ± 2.8	0	0	16.9 ± 3.2	0	0	0.70
AMDR carbohydrate, E%	45–65	-	-	45–65	-	-	45–65	-	-	-
Carbohydrate, E%	47.0 ± 5.9	29	0	44.8 ± 6.1	46	0	45.3 ± 5.9	50	0	0.08
UL added sugar, E%	25	-	-	25	-	-	25	-	-	-
Added sugar, E%	9.8 ± 3.8	100	0	9.4 ± 4.0	96	0	9.1 ± 3.2	100	0	0.71
AMDR, total fat, E%	20–35	-	-	20–35	-	-	20–35	-	-	-
Total fat, E%	35.5 ± 5.0	0	57	36.6 ± 4.6	0	64	36.3 ± 4.7	0	64	0.57
SFA, E%	13.6 ± 2.1	-	-	12.9 ± 1.6	-	-	13.0 ± 2.7	-	-	0.32
MUFA%	12.4 ± 2.2	-	-	13.0 ± 2.1	-	-	13.0 ± 2.1	-	-	0.30
PUFA%	6.7 ± 2.1	-	-	7.8 ± 2.8	-	-	7.4 ± 1.8	-	-	0.08

^a^*n* = 27 for 3 and 6 months post-natal; ^b^ *p*-value for repeated measures ANOVA performed to assess variations in energy and macronutrient intakes across periods. When no dietary reference intake is established for a nutrient, the “-” is used instead of a 0. AMDR: acceptable macronutrient distribution range; EER: estimated energy requirements, calculated with the following formula: 354 − (6.91 × age) + physical activity coefficient × [(9.36 × weight) + (726 × height)], to which an additional 452 kcal were added in the 3rd trimester or an additional 500 kcal were added in the postpartum period if the participant was breastfeeding; UL: upper limit of 25% of the energy provided by added sugar according to the dietary reference intakes. SFA: saturated fatty acids; MUFA: monounsaturated fatty acids; PUFA: polyunsaturated fatty acids.

**Table 3:** Period-specific micronutrient intake from food alone in comparison with dietary reference intakes.

	3rd Trimester of Pregnancy			3 Months Postpartum			6 Months Postpartum			
	Mean ± SD	%Below EAR	%Above UL	Mean ± SD	%Below EAR	%Above UL	Mean ± SD	%Below EAR	%Above UL	Overall *p*-Value ^a^
Vitamin D IU/day	292 ± 128	75	0	252 ± 116	86	0	236 ± 105	93	0	0.06
Iron, mg/day	14.8 ± 3.7	96	0	14.9 ± 3.3	0	0	14.0 ± 3.5	0	0	0.09
Folate, µg DFE/day	518 ± 151	57	-	519 ± 130	36	-	474 ± 125	43	-	0.11
Folic acid, µg/day	136 ± 75	-	0	151 ± 67	-	0	134 ± 66	-	0	0.28
Vitamin B_6_, mg/day	1.8 ± 0.4	32	0	1.8 ± 0.1	36	0	1.7 ± 0.4	50	0	0.13
Magnesium, mg/day	424 ± 79	4	-	399 ± 99	7	-	400 ± 100	11	-	0.12
Vitamin A, µg RAE/day	975 ± 371	7	0	817 ± 317	68	0	752 ± 334	71	0	0.005
Zinc, mg/day	13.8 ± 3.9	7	0	12.2 ± 3.6	32	0	12.4 ± 3.9	36	0	0.06
Calcium, mg/day	1560 ± 453	0	0	1252 ± 386	18	0	1217 ± 377	18	0	0.002
Vitamin C, mg/day	148 ± 73	11	0	109 ± 58	25	0	108 ± 53	43	0	0.03
Thiamin, mg/day	1.8 ± 0.5	4	-	1.8 ± 0.6	7	-	1.7 ± 0.5	14	-	0.01
Vitamin B_12_, µg/day	5.8 ± 2.6	0	-	4.8 ± 1.7	7	-	4.7 ± 1.7	11	-	0.09
Riboflavin, mg/day	2.7 ± 0.7	0	-	2.4 ± 0.5	0	-	2.3 ± 0.6	7	-	0.004
Niacin, mg NE/day	46.1 ± 9.0	0	-	47.0 ± 9.4	0	-	45.9 ± 11.3	0	-	0.70
Pantothenic acid, mg/day	6.9 ± 1.6	-	-	6.4 ± 1.5	-	-	6.4 ± 1.6	-	-	0.18
Phosphorus, mg/day	1768 ± 362	0	0	1614 ± 346	0	0	1549 ± 354	0	0	0.01
Sodium, mg/day	3303 ± 893	-	86	3229 ± 921	-	86	3236 ± 1107	-	79	0.93
Manganese, mg/day	4.3 ± 1.3	-	0	4.1 ± 1.2	-	0	3.9 ± 1.2	-	0	0.17
Selenium, µg/day	136 ± 27	0	0	137 ± 34	0	0	136 ± 49	0	0	0.99
Copper, mg/day	1.6 ± 0.3	0	0	1.5 ± 0.3	4	0	1.5 ± 0.8	4	0	0.50

^a^*p*-value for repeated measures ANOVA performed to assess variations in micronutrient intake across periods. When no EAR or UL was established for a nutrient, the “-” is used instead of a 0. EAR: estimated average requirements; UL: upper intake limit; DFE: dietary folate equivalent; RAE: retinol activity equivalents; NE: niacin equivalent.

**Table 4:** Proportions of vitamin- and mineral-supplement users among participants.

	n (%)			
	3rd Trimester of Pregnancy	3 Months Postpartum	6 Months Postpartum	Overall *p*-Value ^a^
≥1 supplement (all types)	25 (89)	19 (68)	13 (46)	0.003
Number of supplements (all types) taken during each period				
0	3 (11)	9 (32)	15 (54)	0.003
1	21 (75)	14 (50)	11 (39)	0.03
≥2	4 (14)	5 (18)	2 (7)	0.69
Types of supplements most commonly taken				
Multivitamins	23 (92)	16 (84)	12 (92)	0.01
Folic acid supplement	2 (8)	1 (5)	1 (8)	0.77
Vitamin D supplement	2 (8)	2 (11)	1 (8)	0.81
Iron supplement	1 (4)	1 (5)	0 (0)	0.60
Omega-3 supplement (mostly EPA-DHA)	1 (4)	1 (5)	1 (8)	1.00

^a^*p*-value for chi-square tests performed to assess variations in supplement use across periods.

**Table 5:** Period-specific total micronutrient intake (including food sources and supplements) in comparison with dietary reference intakes.

	3rd Trimester of Pregnancy			3 Months Postpartum			6 Months Postpartum			
	Mean ± SD	%Below EAR	%Above UL	Mean ± SD	%Below EAR	%Above UL	Mean ± SD	%Below EAR	%Above UL	Overall *p*-Value ^a^
Vitamin D IU/day	841 ± 776	11	4	684 ± 848	36	4	586 ± 846	57	4	0.001
Iron, mg/day	45.5 ± 32.7	18	39	33.8 ± 18.5	0	25	26.7 ± 15.7	0	14	0.03
Folate, µg DFE/day	1175 ± 993	18	-	1608 ± 2039	18	-	1095 ± 1045	21	-	0.30
Folic acid, µg/day	793 ± 999	-	46	1240 ± 1991	-	61	755 ± 1023	-	46	0.29
Vitamin B_6_, mg/day	6.1 ± 4.2	4	0	14.1 ± 46.9	14	4	3.8 ± 3.7	32	0	0.01
Magnesium, mg/day	474 ± 81	0	0	429 ± 106	7	0	420 ± 106	11	0	0.01
Vitamin A, µg RAE/day	1473 ± 607	4	0	924 ± 356	54	0	838 ± 360	54	0	<0.0001
Zinc, mg/day	23.1 ± 7.3	0	0	19.1 ± 8.6	18	4	17.1 ± 8.1	25	0	0.001
Calcium, mg/day	1779 ± 471	0	4	1415 ± 387	11	0	1330 ± 433	14	0	<0.0001
Vitamin C, mg/day	229 ± 70	4	0	171 ± 86	25	0	151 ± 78	25	0	0.001
Thiamin, mg/day	3.5 ± 1.1	0	-	3.2 ± 1.6	0	-	2.5 ± 1.2	7	-	0.001
Vitamin B_12_, µg/day	11.2 ± 5.7	0	-	8.8 ± 6.1	4	-	7.2 ± 4.6	7	-	0.002
Riboflavin, mg/day	4.5 ± 1.5	0	-	3.8 ± 1.6	0	-	3.2 ± 1.4	4	-	0.0002
Niacin, mg NE/day	61.7 ± 12.2	0	0	58.1 ± 15.2	0	4	53.4 ± 15.8	0	0	0.01
Pantothenic acid, mg/day	11.6 ± 2.9	-	-	9.9 ± 3.4	-	-	8.9 ± 3.6	-	-	0.001
Phosphorus, mg/day	1177 ± 349	0	0	1614 ± 346	0	0	1549 ± 354	0	0	0.01
Sodium, mg/day	3303 ± 893	-	86	3229 ± 921	-	86	3236 ± 1107	-	79	0.93
Manganese, mg/day	5.4 ± 1.2	-	0	4.8 ± 1.4	-	0	4.5 ± 1.1	-	0	0.002
Selenium, µg/day	150 ± 31	0	0	148 ± 38	0	0	145 ± 55	0	0	0.91
Copper, mg/day	2.8 ± 0.9	0	0	2.3 ± 1.1	4	0	2.1 ± 1.1	4	0	0.02

^a^*p*-value for repeated measures ANOVA performed to assess variations in micronutrient intake across periods. When no EAR or UL was established for a nutrient, the “-” is used instead of a 0. EAR: estimated average requirements; UL: upper intake limit; DFE: dietary folate equivalent; RAE: retinol activity equivalents; NE: niacin equivalent.

**Table 6:** Healthy Eating Index total and subscores from late pregnancy to postpartum.

HEI	Score Range	3rd Trimester	3 Months Postpartum	6 Months Postpartum	Overall *p*-Value ^a^
Total	0–100	64.1 ± 12.2	61.2 ± 11.4	60.5 ± 8.3	0.13
Adequacy	0–60	46.4 ± 7.6	44.5 ± 8.3	43.3 ± 7.5	0.08
Total vegetables and fruit	0–10	8.2 ± 2.0	7.1 ± 2.2	6.9 ± 2.4	0.04
Whole fruit	0–5	4.4 ± 1.5	3.6 ± 1.9	3.4 ± 1.7	0.09
Dark green and orange vegetables	0–5	3.3 ± 1.6	3.4 ± 1.5	2.9 ± 1.6	0.25
Total grains products	0–5	4.4 ± 0.7	4.5 ± 0.8	4.2 ± 1.0	0.29
Whole grains	0–5	2.6 ± 1.8	2.7 ± 1.7	2.3 ± 1.9	0.62
Milk and alternatives	0–10	9.5 ± 1.4	8.8 ± 1.9	8.8 ± 2.4	0.08
Meat and alternatives	0–10	8.9 ± 1.6	8.6 ± 2.0	8.5 ± 1.9	0.16
Unsaturated fat	0–10	5.2 ± 3.7	5.8 ± 3.7	6.4 ± 3.3	0.29
Moderation	0–40	17.7 ± 7.2	16.7 ± 6.7	17.2 ± 7.6	0.65
Saturated fats	0–10	2.8 ± 2.6	3.5 ± 2.4	3.7 ± 3.1	0.33
Sodium	0–10	4.6 ± 2.8	4.8 ± 2.9	4.8 ± 3.2	0.93
Other foods	0–20	10.3 ± 5.1	8.4 ± 5.2	8.7 ± 4.8	0.16

^a^*p*-value for repeated measures ANOVA performed to assess variations in HEI-scores across periods.

## Supplementary Materials

The following are available online at https://www.mdpi.com/2072-6643/11/9/2080/s1, Table S1: Components of Canadian adaptation of Healthy Eating Index, range of scores and scoring criteria, Table S2: Period-specific macronutrient intake in comparison with dietary reference intakes.

## References

[1] Kaiser L., Allen L.H. (2008). Position of the American Dietetic Association: Nutrition and lifestyle for a healthy pregnancy outcome. J. Am. Diet. Assoc..

[2] Symonds M.E.R. (2010). Maternal-Fetal Nutrition during Pregnancy and Lactation.

[3] Rolfes S.R., Pinna K., Whitney E. (2002). Understanding Normal and Clinical Nutrition.

[4] Boghossian N.S., Yeung E.H., Lipsky L.M., Poon A.K., Albert P.S. (2013). Dietary patterns in association with postpartum weight retention. Am. J. Clin. Nutr..

[5] Flegal K.M., Graubard B.I., Williamson D.F., Gail M.H. (2007). Cause-specific excess deaths associated with underweight, overweight, and obesity. JAMA.

[6] Rong K., Yu K., Han X., Szeto I.M., Qin X., Wang J., Ning Y., Wang P., Ma D. (2015). Pre-pregnancy BMI, gestational weight gain and postpartum weight retention: A meta-analysis of observational studies. Public Health Nutr..

[7] Tahir M.J., Haapala J.L., Foster L.P., Duncan K.M., Teague A.M., Kharbanda E.O., McGovern P.M., Whitaker K.M., Rasmussen K.M., Fields D.A. (2019). Higher Maternal Diet Quality during Pregnancy and Lactation Is Associated with Lower Infant Weight-For-Length, Body Fat Percent, and Fat Mass in Early Postnatal Life. Nutrients.

[8] Wennberg A.L., Isaksson U., Sandstrom H., Lundqvist A., Hornell A., Hamberg K. (2016). Swedish women’s food habits during pregnancy up to six months post-partum: A longitudinal study. Sex. Reprod. Healthc..

[9] Olson C.M. (2005). Tracking of food choices across the transition to motherhood. J. Nutr. Educ. Behav..

[10] George G.C., Hanss-Nuss H., Milani T.J., Freeland-Graves J.H. (2005). Food choices of low-income women during pregnancy and postpartum. J. Am. Diet. Assoc..

[11] Wiltheiss G.A., Lovelady C.A., West D.G., Brouwer R.J., Krause K.M., Ostbye T. (2013). Diet quality and weight change among overweight and obese postpartum women enrolled in a behavioral intervention program. J. Acad. Nutr. Diet..

[12] George G.C., Milani T.J., Hanss-Nuss H., Freeland-Graves J.H. (2005). Compliance with dietary guidelines and relationship to psychosocial factors in low-income women in late postpartum. J. Am. Diet. Assoc..

[13] Savard C., Lemieux S., Carbonneau E., Provencher V., Gagnon C., Robitaille J., Morisset A.S. (2019). Trimester-Specific Assessment of Diet Quality in a Sample of Canadian Pregnant Women. Int. J. Environ. Res. Public Health.

[14] Savard C., Lemieux S., Weisnagel S.J., Fontaine-Bisson B., Gagnon C., Robitaille J., Morisset A.S. (2018). Trimester-Specific Dietary Intakes in a Sample of French-Canadian Pregnant Women in Comparison with National Nutritional Guidelines. Nutrients.

[15] Jacques S., Lemieux S., Lamarche B., Laramee C., Corneau L., Lapointe A., Tessier-Grenier M., Robitaille J. (2016). Development of a Web-Based 24-h Dietary Recall for a French-Canadian Population. Nutrients.

[16] Lafreniere J., Laramee C., Robitaille J., Lamarche B., Lemieux S. (2018). Assessing the relative validity of a new, web-based, self-administered 24 h dietary recall in a French-Canadian population. Public Health Nutr..

[17] Savard C., Lemieux S., Lafreniere J., Laramee C., Robitaille J., Morisset A.S. (2018). Validation of a self-administered web-based 24-hour dietary recall among pregnant women. BMC Pregnancy Childbirth.

[18] Garriguet D. (2009). Diet quality in Canada. Health Rep..

[19] Kennedy E.T., Ohls J., Carlson S., Fleming K. (1995). The Healthy Eating Index: Design and applications. J. Am. Diet. Assoc..

[20] Moran L.J., Sui Z., Cramp C.S., Dodd J.M. (2013). A decrease in diet quality occurs during pregnancy in overweight and obese women which is maintained post-partum. Int. J. Obes. (Lond.).

[21] Shin D., Bianchi L., Chung H., Weatherspoon L., Song W.O. (2014). Is gestational weight gain associated with diet quality during pregnancy?. Matern. Child Health J..

[22] Tsigga M., Filis V., Hatzopoulou K., Kotzamanidis C., Grammatikopoulou M.G. (2011). Healthy Eating Index during pregnancy according to pre-gravid and gravid weight status. Public Health Nutr..

[23] (2007). Eating Well with Canada’s Food Guide.

[24] Health Canada Licensed Natural Health Product Database. https://health-products.canada.ca/lnhpd-bdpsnh/index-eng.jsp.

[25] Chandonnet N., Saey D., Almeras N., Marc I. (2012). French Pregnancy Physical Activity Questionnaire compared with an accelerometer cut point to classify physical activity among pregnant obese women. PLoS ONE.

[26] Chasan-Taber L., Schmidt M.D., Roberts D.E., Hosmer D., Markenson G., Freedson P.S. (2004). Development and validation of a Pregnancy Physical Activity Questionnaire. Med. Sci. Sports Exerc..

[27] Craig C.L., Marshall A.L., Sjostrom M., Bauman A.E., Booth M.L., Ainsworth B.E., Pratt M., Ekelund U., Yngve A., Sallis J.F. (2003). International physical activity questionnaire: 12-country reliability and validity. Med. Sci. Sports Exerc..

[28] Otten J.J., Hellwig J.P., Meyers L.D. (2006). Dietary Reference Intakes: The Essential Guide to Nutrient Requirements.

[29] Institute of Medicine (US) Subcommittee on Interpretation and Uses of Dietary Reference Intakes, Institute of Medicine (US) Standing Committee on the Scientific Evaluation of Dietary Reference Intakes (2000). Application of DRIs for Group Diet Assessment. DRI Dietary Reference Intakes: Applications in Dietary Assessment.

[30] Talai Rad N., Ritterath C., Siegmund T., Wascher C., Siebert G., Henrich W., Buhling K.J. (2011). Longitudinal analysis of changes in energy intake and macronutrient composition during pregnancy and 6 weeks post-partum. Arch. Gynecol. Obstet..

[31] Derbyshire E., Davies G.J., Costarelli V., Dettmar P.W. (2009). Habitual micronutrient intake during and after pregnancy in Caucasian Londoners. Matern. Child Nutr..

[32] Horan M.K., McGowan C.A., Gibney E.R., Byrne J., Donnelly J.M., McAuliffe F.M. (2016). Maternal Nutrition and Glycaemic Index during Pregnancy Impacts on Offspring Adiposity at 6 Months of Age—Analysis from the ROLO Randomised Controlled Trial. Nutrients.

[33] Lindsay K.L., Heneghan C., McNulty B., Brennan L., McAuliffe F.M. (2015). Lifestyle and dietary habits of an obese pregnant cohort. Matern. Child Health J..

[34] Moran L.J., McNaughton S.A., Sui Z., Cramp C., Deussen A.R., Grivell R.M., Dodd J.M. (2018). The characterisation of overweight and obese women who are under reporting energy intake during pregnancy. BMC Pregnancy Childbirth.

[35] Brassard D., Laramee C., Corneau L., Begin C., Belanger M., Bouchard L., Couillard C., Desroches S., Houle J., Langlois M.F. (2018). Poor Adherence to Dietary Guidelines among French-Speaking Adults in the Province of Quebec, Canada: The PREDISE Study. Can. J. Cardiol..

[36] Wilson R.D., Wilson R.D., Audibert F., Brock J.A., Carroll J., Cartier L., Gagnon A., Johnson J.A., Langlois S., Murphy-Kaulbeck L. (2015). Pre-Conception Folic Acid and Multivitamin Supplementation for the Primary and Secondary Prevention of Neural Tube Defects and Other Folic Acid-Sensitive Congenital Anomalies. J. Obstet. Gynaecol. Can..

[37] Bassett-Gunter R.L., Levy-Milne R., Naylor P.J., Symons Downs D., Benoit C., Warburton D.E., Blanchard C.M., Rhodes R.E. (2013). Oh baby! Motivation for healthy eating during parenthood transitions: A longitudinal examination with a theory of planned behavior perspective. Int. J. Behav. Nutr. Phys. Act..

[38] Hanson M.A., Bardsley A., De-Regil L.M., Moore S.E., Oken E., Poston L., Ma R.C., McAuliffe F.M., Maleta K., Purandare C.N. (2015). The International Federation of Gynecology and Obstetrics (FIGO) recommendations on adolescent, preconception, and maternal nutrition: “Think Nutrition First”. Int. J. Gynaecol. Obstet..

