# Supplementary material for: Tracking of Dietary Intake and Diet Quality from Late Pregnancy to the Postpartum Period
Source: Nutrients. 2019 Sep 3;11(9):2080. doi: 10.3390/nu11092080 (PMC6769665; doi:10.3390/nu11092080)
Supplement: Supplementary file 1 [file nutrients-11-02080-s001.pdf]

**Table S1.** Components of Canadian adaptation of Healthy Eating Index, range of scores and scoring criteria.

| Component | Scoring criteria (daily servings) * | |
|---|---|---|
| | **Minimum score** | **Maximum score** |
| **Adequacy** † | | |
| Total vegetables and fruit | 0 | 4 to 10 servings |
| Whole fruit | 0 | 0.8 to 2.1 servings |
| Dark green and orange vegetables | 0 | 0.8 to 2.1 servings |
| Total grain products | 0 | 3 to 8 servings |
| Whole grains | 0 | 1.5 to 4 servings |
| Milk and alternatives | 0 | 2 to 4 servings |
| Meat and alternatives | 0 | 1 to 3 servings |
| Unsaturated fats | 0 | 30 to 45 grams/day § |
| **Moderation** ‡ | | |
| Saturated fats | ≥15% of total energy intake | ≤7% of total energy intake |
| Sodium | Twice the UL | AI or less (≤1500 mg) |
| Other foods | ≥40% of total energy intake | ≤5% of total energy intake |

Adapted from Garriguet D. (2009); UL, Upper Intake limit; AI, Adequate intake. * according to age and sex, as specified in Canada's Food Guide. † for adequacy components, 0 points for minimum intake or less, 5, 10 or 20 for maximum intake or more, and proportional for amounts between minimum and maximum. ‡ for moderation components, 10 or 20 points for minimum intake or less, 0 points for maximum intake or more, and proportional for amounts between minimum and maximum. § Refers only to the amount of unsaturated fats added directly to food during cooking or through salad dressings and does not represents total dietary intake of unsaturated fats.

**Table S2.** Period-specific macronutrient intake in comparison with dietary reference intakes.

| | 3rd trimester of pregnancy | | | 3 months postpartum | | | 6 months postpartum | | | |
|---|---|---|---|---|---|---|---|---|---|---|
| | **Mean ± SD** | **%Below EPR or AI** | **%Above EPR or AI** | **Mean ± SD** | **%Below EPR or AI** | **%Above EPR or AI** | **Mean ± SD** | **%Below EPR or AI** | **%Above EPR or AI** | **Overall *p*-Value [a]** |
| EPR, g/day | 95.7 ± 9.4 | - | - | 83.9 ± 10.1 | - | - | 80.2 ± 12.6 | - | - | - |
| Protein, g/day | 100 ± 19 | 36 | 64 | 97.1 ± 18.9 | 25 | 75 | 93.2 ± 21.6 | 25 | 75 | 0.22 |
| Carbohydrate, g/day | 283 ± 64 | - | - | 266 ± 68 | - | - | 259 ± 65 | - | - | 0.11 |
| Total fat, g/day | 91.9 ± 22.7 | - | - | 93.8 ± 24.5 | - | - | 90.5 ± 23.5 | - | - | 0.75 |
| Dietary fiber, g/day | 24.6 ± 5.6 | 89 | 11 | 22.0 ± 4.5 | 93 | 7 | 21.3 ± 6.6 | 89 | 11 | 0.01 |
| ω-6 Linoleic acid, g/day | 9.6 ± 5.6 | - | - | 11.1 ± 5.6 | - | - | 11.1 ± 5.3 | - | - | 0.23 |
| ω-3 Linolenic acid, g/day | 2.0 ± 1.0 | - | - | 2.1 ± 1.0 | - | - | 2.1 ± 0.8 | - | - | 0.84 |
| Cholesterol, mg/day | 297 ± 105 | - | - | 293 ± 120 | - | - | 306 ± 128 | - | - | 0.87 |

[a] *p*-value for repeated measures ANOVA performed to assess variations in macronutrient intake across periods. When no dietary reference intake was established for a nutrient, the "-" is used instead of a 0. EPR: estimated protein requirement (1.1 g/kg of pre-pregnancy weight + 25 g for the third trimester and 1.3 g/kg for participants who breastfed or 0.8 g/kg for those who didn't in the postpartum period). AI: adequate intake (14 g of fiber/1000 kcal consumed).
